# Hand-Washing Practices among Adolescents Aged 12–15 Years from 80 Countries

**DOI:** 10.3390/ijerph18010138

**Published:** 2020-12-27

**Authors:** Lee Smith, Laurie Butler, Mark A Tully, Louis Jacob, Yvonne Barnett, Guillermo F. López-Sánchez, Rubén López-Bueno, Jae Il Shin, Daragh McDermott, Briona A. Pfeifer, Damiano Pizzol, Ai Koyanagi

**Affiliations:** 1 The Cambridge Centre for Sport and Exercise Sciences, Anglia Ruskin University, Cambridge CB1 1PT, UK; brionapfeifer@gmail.com; 2 Faculty of Science and Engineering, Anglia Ruskin University, Cambridge CB1 1PT, UK; laurie.butler@anglia.ac.uk; 3 Institute of Mental Health Sciences, School of Health Sciences, Ulster University, Newtownabbey BT37 0QB, UK; m.tully@ulster.ac.uk; 4 Faculty of Medicine, University of Versailles Saint-Quentin-en-Yvelines, 78180 Montigny-le-Bretonneux, France; louis.jacob.contacts@gmail.com; 5 Faculty of Science and Technology, Anglia Ruskin University, Cambridge CB1 1PT, UK; yvonne.barnett@aru.ac.uk; 6 Vision and Eye Research Institute, School of Medicine, Faculty of Health, Education, Medicine and Social Care, Anglia Ruskin University-Cambridge Campus, Cambridge CB1 1PT, UK; guillermo.lopez-sanchez@aru.ac.uk; 7 Department of Physical Medicine and Nursing, University of Zaragoza, 50009 Zaragoza, Spain; rlopezbu@unizar.es; 8 Department of Pediatrics, Yonsei University College of Medicine, Yonsei-ro 50, Seodaemun-gu, C.P.O. Box 8044, Seoul 120-752, Korea; SHINJI@yuhs.ac; 9 School of Psychology and Sport, Anglia Ruskin University, Cambridge CB1 1PT, UK; daragh.mcdermott@anglia.ac.uk; 10 Italian Agency for Development Cooperation, Khartoum 79371, Sudan; damianopizzol8@gmail.com; 11 Research and Development Unit, Parc Sanitari Sant Joan de Déu, CIBERSAM, ICREA, Sant Boi de Llobregat, 08830 Barcelona, Spain

**Keywords:** hand-washing, hygiene, epidemiology, adolescents, poverty, multi-country study

## Abstract

The objectives were to (1) assess the prevalence of hand-washing practices across 80 countries and (2) assess frequency of hand-washing practice by economic status (country income and severe food insecurity), in a global representative sample of adolescents. Cross-sectional data from the Global School-based Student Health Survey 2003–2017 were analyzed. Data on age, sex, hand-washing practices in the past 30 days, and severe food insecurity (i.e., proxy of socioeconomic status) were self-reported. Multivariable logistic regression and meta-analysis with random effects based on country-wise estimates were conducted to assess associations. Adolescents (*n* = 209,584) aged 12–15 years [mean (SD) age 13.8 (1.0) years; 50.9% boys] were included in the analysis. Overall, the prevalence of hand-washing practices were as follows: never/rarely washing hands before eating (6.4%), after using toilet (5.6%), or with soap (8.8%). The prevalence of never/rarely washing hands after using the toilet (10.8%) or with soap (14.3%) was particularly high in low-income countries. Severe food insecurity was associated with 1.34 (95%CI = 1.25–1.43), 1.61 (95%CI = 1.50–1.73), and 1.44 (95%CI = 1.35–1.53) times higher odds for never/rarely washing hands before eating, after using the toilet, and with soap, respectively. A high prevalence of inadequate hand washing practices was reported, particularly in low-income countries and those with severe food insecurity. In light of the present COVID-19 pandemic and the rapid expansion being observed in low- and middle-income locations, interventions that disseminate good hand-washing practices are urgently required. Such interventions may also have cross-over benefits in relation to other poor sanitation-related diseases.

## 1. Introduction

Hand-washing with soap has been found to be an effective prevention strategy against the contraction of infectious diseases, including respiratory infections and gastrointestinal diseases. For example, findings from meta-analyses suggest that hand-washing with soap can reduce respiratory infections by 21% to 23% [1,2] and diarrheal disease by 23% to 48% [3,4,5].

In March 2020, the World Health Organization (WHO) declared the COVID-19 outbreak a global pandemic. COVID-19 is caused by SARS-CoV-2, a variant of coronavirus. As of 17 April 2020 (10:00 am CET), more than 2,160,170 cases have been diagnosed globally, with over 145,593 directly reported fatalities globally [6]. COVID-19 is a respiratory virus that is transmitted by large respiratory droplets and direct contact with infected secretions. One very important measure to prevent contraction is to wash hands with soap regularly and this is at the heart of current public health guidance, amongst other strategies (e.g., self-isolating and social distancing). The WHO states “regularly and thoroughly clean your hands with an alcohol-based hand rub or wash them with soap and water” [7]. 

However, despite this, limited data exists on the prevalence of hand-washing in different countries. One systematic review investigated the global prevalence of hand washing with soap after potential fecal contact and found that this stood at approximately 26%. Moreover, in regions with high access to hand-washing facilities, hand-washing with soap was performed by about 51%, and in regions with more limited access, by about 22% after events of potential fecal contact [8]. Finally, the review concluded that important gaps exist for country-representative data on presence of designated hand-washing facilities and on hand-washing behavior at household level, for all regions of the world and particularly for high-income countries (HICs) [8]. Before interventions on hand-washing practice can be implemented with confidence to prevent the spread of infection, such as in the case of SARS-CoV-2, levels of hand-washing practices by country are required, in order to appropriately inform public health information campaigns. 

In addition, to promote effective hand-washing practices, vulnerable populations need to be identified. Previous studies have shown that those from poorer socio-economic backgrounds engage in particularly inadequate hand-washing practices. This may be explained by lack of adequate washing facilities or soap, or lack of knowledge on the importance of maintaining hygiene. One study in 6971 children participants residing in urban Bangladesh found that hand-washing indicators were strongly influenced by socio-economic status. For example, those in the poorest wealth category washed hands with soap significantly less before eating and after defecation when compared to those in higher wealth categories [9]. In another study of 800 Kenyan households those with the lowest level of education washed their hands markedly less than the majority of households [10]. Other studies from low- and middle-income countries (LMICs) have found similar findings [11,12]. However, studies investigating the association between socio-economic status and hand washing from a global perspective including HICs are lacking. There are markedly different socio-cultural norms, occupational and family structures, societal norms, environmental features (e.g., housing types, availability of hand washing facilities) between settings (e.g., LMICs and HICs). Therefore, there is a need for context-specific research [13]. Moreover, multi-country studies, which include both LMICs and HICs, are important, as they provide a platform to investigate between-country differences utilizing standardized data. 

Furthermore, adolescents in particular may play an important role in the transmission of infectious diseases during pandemic times owing to high levels of risk taking behavior [14]. Adolescents may be less likely to follow government guidance, such as in the case of COVID-19, making good hand-washing practices even more essential in this population to prevent the spread and acquisition of infectious disease [14]. However, there are currently only a few studies which have specifically focused on hand-washing practices among adolescents.

Given the above-mentioned gaps in the literature, the present study aimed to (1) assess the prevalence of hand-washing practices across 80 countries and (2) assess frequency of hand-washing practice by socio-economic status (country income and severe food insecurity), in a globally representative sample of adolescents.

## 2. Methods

Publicly available data from the GSHS were analyzed. Details of this survey can be found at http://www.who.int/chp/gshs and http://www.cdc.gov/gshs. Briefly, the GSHS was jointly developed by the WHO and the US Centers for Disease Control and Prevention (CDC), and other UN allies. The core aim of this survey was to assess and quantify risk and protective factors of major non-communicable diseases. The survey draws content from the CDC Youth Risk Behavior Survey (YRBS) for which test-retest reliability has been established [15]. The survey used a standardized two-stage probability sampling design for the selection process within each participating country. For the first stage, schools were selected with probability proportional to size sampling. The second stage involved the random selection of classrooms which included students aged 13–15 years within each selected school. All students in the selected classrooms were eligible to participate in the survey regardless of age. Data collection was performed during one regular class period. The questionnaire was translated into the local language in each country and consisted of multiple choice response options; students recorded their response on computer scannable sheets. All GSHS surveys were approved, in each country, by both a national government administration (most often the Ministry of Health or Education) and an institutional review board or ethics committee. Student privacy was protected through anonymous and voluntary participation, and informed consent was obtained as appropriate from the students, parents and/or school officials. Data were weighted for non-response and probability selection.

From all publicly available data, we selected all nationally representative datasets that included the variables used in the current analysis. If there were more than two datasets from the same country, we chose the most recent dataset. A total of 80 countries were included in the current study, and consisted of 12 low-income, 34 lower middle-income, 21 upper middle-income, and 13 high-income countries based on the World Bank classification at the time of the survey. The characteristics of each country or survey are provided in Table 1. For the included countries, the survey was conducted between 2003 and 2017.

### 2.1. Hand-Washing Practices

Three questions about hand-washing practices in the past 30 days were asked: (a) how often did you wash your hand before eating? (b) how often did you wash your hands after using the toilet or latrine?; and (c) how often did you use soap when washing your hands? Each of these questions had as answer options: ‘Never’, ‘Rarely’, ‘Sometimes’, ‘Most of the time’, and ‘Always’. In line with a previous GSHS publication, we dichotomized this variable as ‘Never/rarely’ (Coded 1) and others (Coded 0) [16].

### 2.2. Food Insecurity (Proxy of Socioeconomic Status)

Since the GSHS does not have a question on socioeconomic status, we used food insecurity as a proxy of this condition [17]. Food insecurity was assessed by the question “During the past 30 days, how often did you go hungry because there was not enough food in your home?” with answer options ‘Never’, ‘Rarely’, ‘Sometimes’, ‘Most of the time’, and ‘Always’. As in a previous GSHS study, we considered those who answered ‘Most of the time’ or ‘Always’ as having severe food insecurity [18].

### 2.3. Statistical Analysis

Statistical analyses were performed with Stata 14.1 (Stata Corp LP, College station, TX, USA). The analysis was restricted to those aged 12–15 years as most students were within this age group while information on exact age outside of this age range was not available. The prevalence of hand-washing practices was calculated using the overall sample, and by country or country-income level. The associations between severe food insecurity and each type of hand-washing practice were assessed by country-wise multivariable logistic regression analysis adjusting for age and sex. Pooled estimates were obtained by meta-analysis with random effects based on country-wise estimates. In order to assess the level of between-country heterogeneity in the association between severe food insecurity and hand-washing practices, we also calculated the Higgins’s I2 which represents the degree of heterogeneity that is not explained by sampling error. I2 values of 25%, 50%, and 75% are often considered low, moderate, and high level of heterogeneity, respectively [19]. Sampling weights and the clustered sampling design of the surveys were taken into account in all analyses. Results from the logistic regression analyses are presented as odds ratios (ORs) with 95% confidence intervals (CIs).

## 3. Results

**Figure 1:**
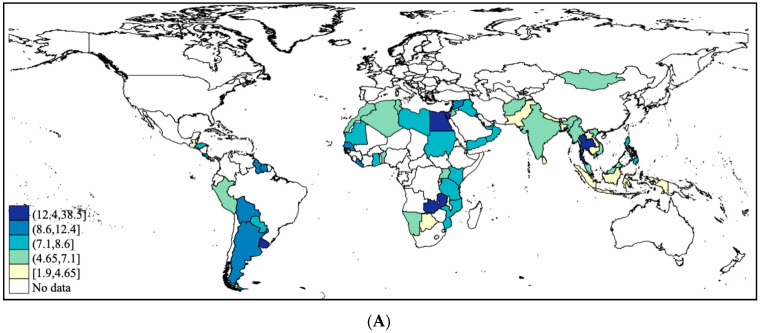

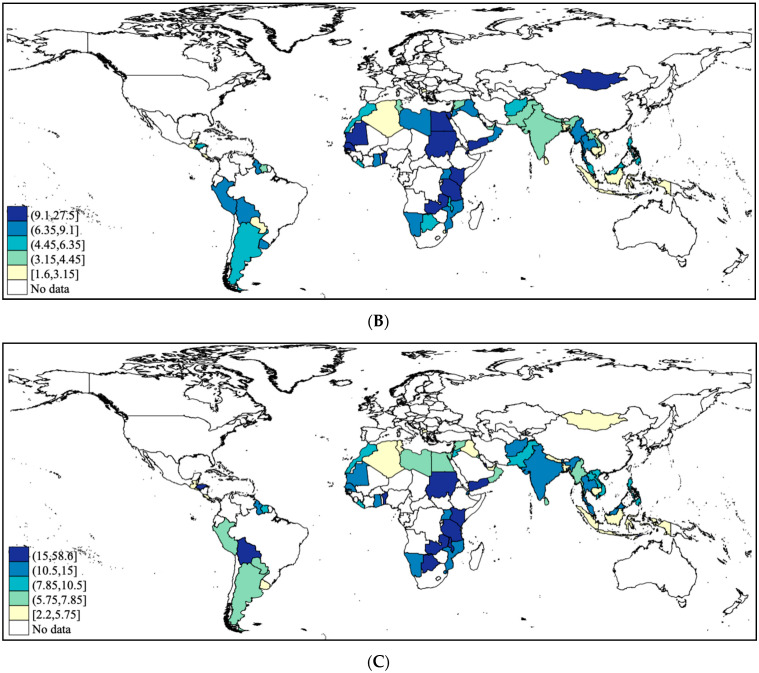
Prevalence of never/rarely washing hands before eating, after using toilet, and with soap by country. (**A**) Prevalence of adolescents who never or rarely wash hands before eating; (**B**) Prevalence of adolescents who never or rarely wash hands after using the toilet; (**C**) Prevalence of adolescents who rarely or never use soap when washing hands.

**Figure 2:**
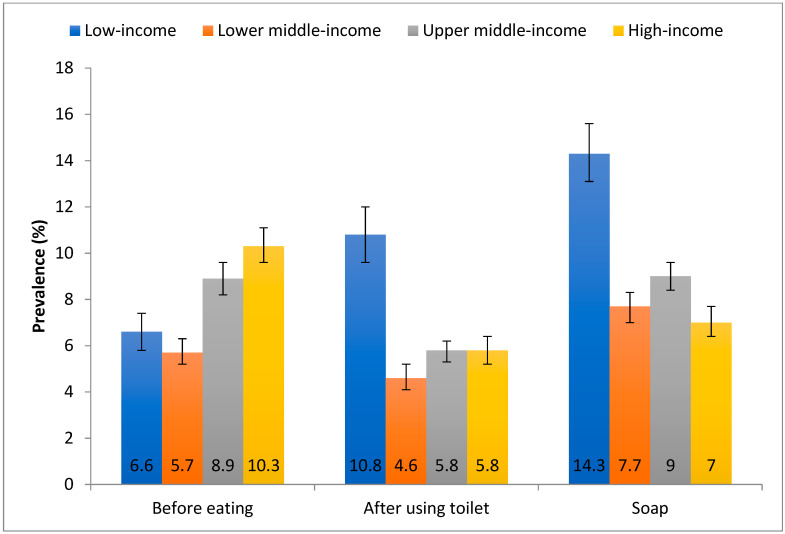
Prevalence of never/rarely washing hands before eating, after using toilet, and with soap by country-income level. Bars denote 95% confidence interval.

**Figure A1:**
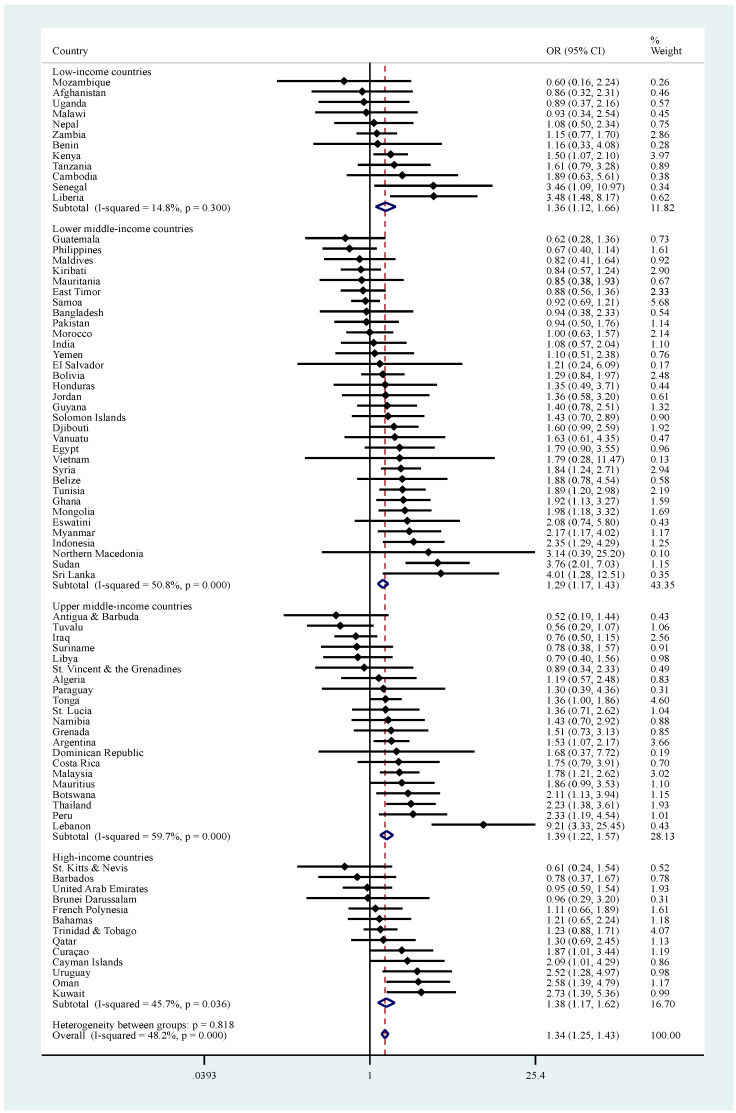
Country-wise association between severe food insecurity and never/rarely washing hands before eating estimated by multivariable logistic regression adjusted for age and sex; Abbreviation: OR Odds ratio; CI Confidence interval; overall estimates were calculated by meta-analysis with random effects.

**Figure A2:**
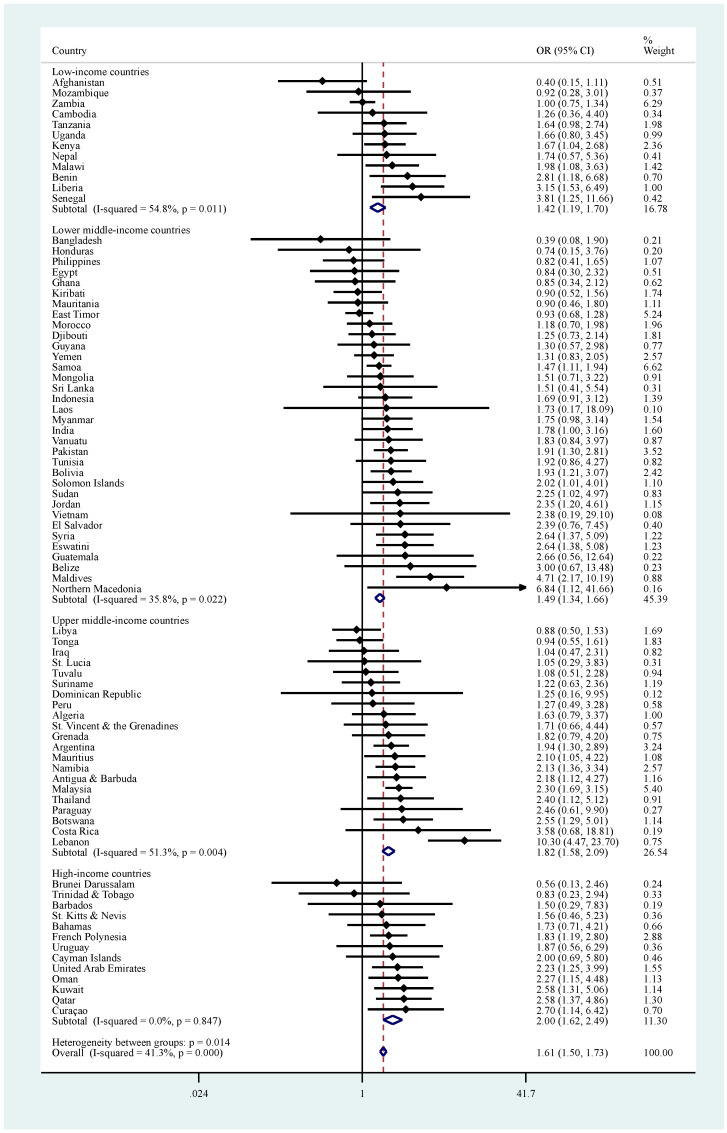
Country-wise association between severe food insecurity and never/rarely washing hands after using toilet estimated by multivariable logistic regression adjusted for age and sex; Abbreviation: OR Odds ratio; CI Confidence interval; overall estimates were calculated by meta-analysis with random effects.

**Figure A3:**
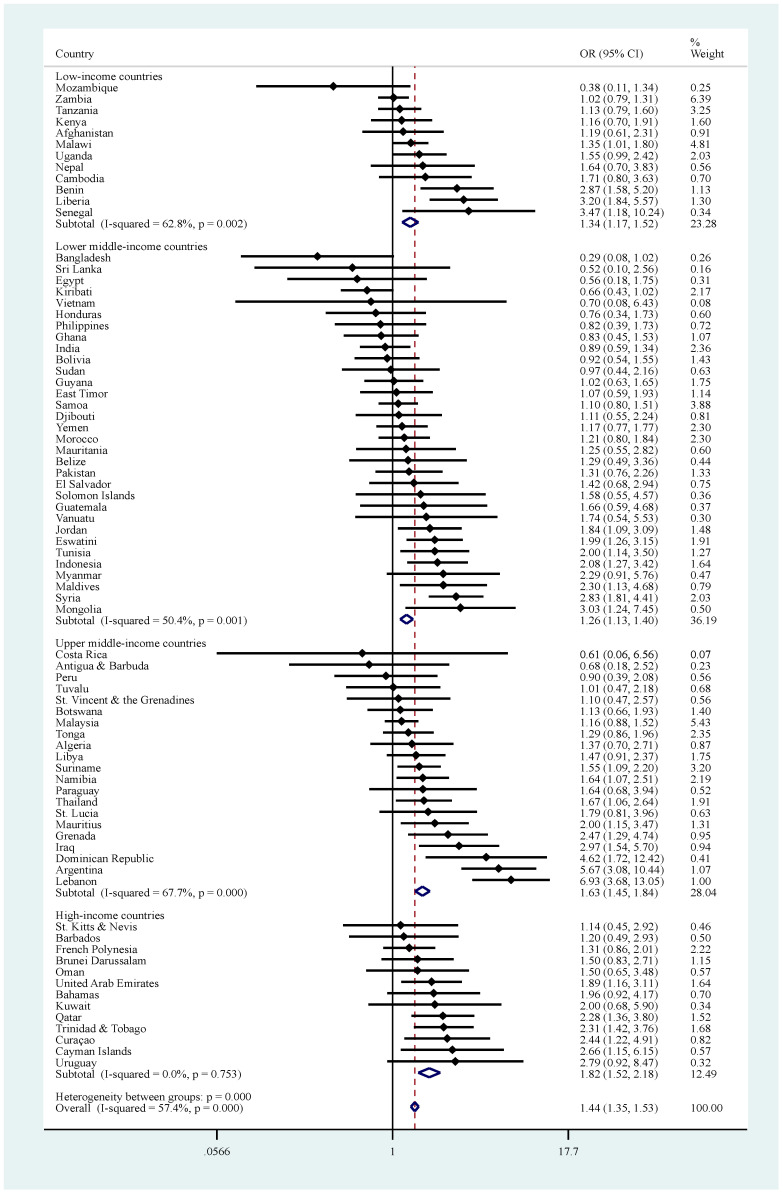
Country-wise association between severe food insecurity and never/rarely using soap when washings hands estimated by multivariable logistic regression adjusted for age and sex; Abbreviation: OR Odds ratio; CI Confidence interval; overall estimates were calculated by meta-analysis with random effects.

A total of 209,584 adolescents aged 12–15 years [mean (SD) age 13.8 (1.0) years; 50.9% boys] were included in the analysis. Overall, the prevalence of hand-washing practices was as follows: never/rarely washing hands before eating (6.4%), after using toilet (5.6%), and with soap (8.8%). The country-wise prevalence is shown in Table 2 and Figure 1. The prevalence of never/rarely washing hands before eating was highest in Tuvalu (38.5%), followed by East Timor (20.9%), and Kiribati (20.6%). In terms of this figure for never/rarely washing hands after using the toilet, the highest figures were observed in East Timor (27.5%), Mauritania (24.4%), and Tuvalu (17.7%), with a high prevalence generally in Africa. As for never or rarely using soap when washing hands, Honduras had the highest prevalence (58.6%), followed by Sudan (20.9%), and Zambia (20.8%), with most countries with the highest prevalence being found in Sub-Saharan Africa. The prevalence of hand-washing practices by country-income level is shown in Figure 2. The prevalence of never or rarely washing hands before eating was higher in high- and upper middle-income countries, while that of never/rarely washing hands after using the toilet and with soap was particularly high in low-income countries. The associations between severe food insecurity and hand-washing practices assessed by meta-analysis based on country-wise estimates are shown in Table 3. Severe food insecurity was associated with 1.34 (95% CI = 1.25–1.43) times higher odds for never/rarely washing hands before eating overall, with the estimates being similar between different country-income levels. The corresponding figure for never/rarely washing hands after using the toilet was 1.61 (95%CI = 1.50–1.73), with higher ORs being observed in countries with higher income. As for never/rarely using soap when washing hands, this figure was 1.44 (95%CI = 1.35–1.53) with the highest ORs being observed in upper middle- and high-income countries. A moderate level of heterogeneity was observed for all overall estimates. The country-wise estimates from which these estimates were derived can be found in Figure A1, Figure A2 and Figure A3 of the Appendix A.

## 4. Discussion

In this large sample of adolescents aged 12–15 years across 80 countries, prevalence of poor hand-washing practices was found to be high: never/rarely washing hands before eating (6.4%), after using toilet (5.6%), and with soap (8.8%). The highest prevalence of never/rarely washing hands after using the toilet and with soap were found in low-income countries. However, the prevalence of never/rarely washing hands before eating was higher in upper middle-income countries and HICs. Generally, those from a low socio-economic status (using severe food insecurity as a proxy) were less likely to engage in good hand washing practices. Findings were similar for each hand-washing scenario (i.e., before eating and after using the toilet, or using soap).

Findings from the present study supports previous literature which has reported a low prevalence of good hand-washing practices particularly in LMICs [9,10,11,12]. This may be owing to lack of knowledge relating to benefits and how to appropriately wash hands. For example, in one study carried out on 90 health care professionals in Northeast Ethiopia, 36.1% had no knowledge of good hand-washing practices [20]. Moreover, poor hand-washing practices may be due to a lack of hand washing facilities, access to clean water supplies or soap in low-income countries [21]. Indeed, in sub-Saharan Africa, 63 percent of people in urban areas–258 million people–lack access to hand washing, according to the UNICEF figures. In central and south Asia this figure is 22 percent, or 153 million people [22]. However, the finding that hand-washing before eating was more common in lower income countries points to the fact that not all hand-washing practices may be linked with poverty. Although the reason for this finding is unknown, it may be explained by the fact that eating with hands is common in some LMICs and it is considered important to wash hands before eating in these settings.

Although the first confirmed COVID-19 cases occurred later in West Africa than in Europe, once these first cases were confirmed the expansion in the number of confirmed COVID-19 was rapid [23]. Based on our findings that the prevalence of never/rarely washing hands after using the toilet or with soap was particularly high in low-income countries and in particular, Sub-Saharan Africa, it is possible that this may have partly expedited the spread of the SARS-Cov-2 in these locations and potentially other contemporaneous locations. Therefore, a strong dissemination of good hand-washing practices especially in low-income countries may help curb the rapid expansion of COVID-19 cases. In addition, such a message may also have a cross-over effect, that is, we may observe reductions in other diseases linked to poor sanitation conditions such as pneumonia, gastroenteritis, diarrhea, dysentery, hepatitis A, cholera, typhoid, polio and skin infection. 

The present study found that severe food insecurity (a proxy for low socio-economic status) was significantly associated with poor hand-washing practices across LMICs and HICs. It is likely that this is due to similar reasons that explain a low prevalence of good hand-washing practice in LMICs, that is lack of knowledge and availability of appropriate facilities. Of note, severe food insecurity was associated with never/rarely washing hands after using the toilet or with soap more strongly in upper middle-income countries and HICs than countries with lower income pointing to the fact that, even within wealthier nations, individuals with lower levels of socioeconomic status are more likely to engage in inadequate hygiene and are possibly at a higher risk for various types of infections.

Clear strengths of this study include the large global sample of adolescents from 80 countries and the inclusion of high-, middle-, and low-income countries. However, finding must be interpreted in light of the study limitations. First, the study relied on self-reported data, thus some degree of bias may exist. Second, our study only included adolescents who attend school, and the results may therefore not be generalizable to all adolescents in the respective countries especially in countries where school attendance rates are low. Third, we did not have information on whether the student was not able to engage in adequate handwashing practices due to lack of facilities or whether this was due to personal choice. Given that these two different underlying reasons may require different interventions, future studies should also investigate the exact reason why some students do not engage in adequate handwashing practices. Finally, data were collected between 2003 and 2017 and thus, our results may not reflect the current situation in some countries. 

## 5. Conclusions

In conclusion, in this large representative global sample of adolescents, a low prevalence of good hand-washing practices was reported, particularly in low-income countries, and those with a low socio-economic status regardless of country-income level. In light of the present COVID-19 pandemic and the rapid expansion globally, interventions that disseminate good hand-washing practices and, ideally, provision of soap in areas in need are urgently required. Moreover, such a message may also have a cross-over effect, that is, we may observe reductions in other diseases linked to poor sanitation conditions such as pneumonia, gastroenteritis, diarrhea, dysentery, hepatitis A, cholera, typhoid, polio and skin infection. Our data show that some good hand-washing practices (e.g., washing hands before eating) may be less common in HICs, and that those with severe food insecurity in HICs have particularly high odds for never/rarely washing hands after using the toilet or with soap. Thus, interventions to promote effective hand-washing to reduce risk for various types of infections are needed worldwide regardless of country-income level and focusing on the socially underprivileged within a country may be important.

## Figures and Tables

**Table 1:** Sample characteristics.

Country-Income	Country	Year	Response Rate (%)	N ^a^	Male (%)	Age (Years) Mean (SD)
Low	Afghanistan	2014	79	1493	53.4	14.0 (0.9)
	Benin	2016	78	717	65.6	14.2 (0.9)
	Cambodia	2013	85	1812	48.4	14.1 (0.8)
	Kenya	2003	84	2971	47.5	13.9 (1.0)
	Liberia	2017	71	541	50.1	14.0 (0.9)
	Malawi	2009	94	2224	51.5	14.0 (0.8)
	Mozambique	2015	80	668	49.6	14.1 (0.8)
	Nepal	2015	69	4616	47.3	13.8 (1.0)
	Senegal	2005	60	2666	60.2	13.9 (1.0)
	Tanzania	2014	87	2615	46.8	13.6 (1.0)
	Uganda	2003	69	1904	47.4	14.3 (0.8)
	Zambia	2004	70	1365	50.3	13.9 (1.0)
Low middle	Bangladesh	2014	91	2753	63.4	14.0 (0.8)
	Belize	2011	88	1600	48.4	13.6 (1.1)
	Bolivia	2012	88	2804	49.7	14.0 (0.9)
	Djibouti	2007	83	962	59.5	14.3 (0.8)
	East Timor	2015	79	1631	46.3	14.1 (1.0)
	Egypt	2011	85	2364	49.2	13.5 (0.9)
	El Salvador	2013	88	1615	50.6	14.0 (0.9)
	Eswatini	2013	97	1318	39.1	14.1 (0.8)
	Ghana	2012	82	1110	49.1	13.8 (1.0)
	Guatemala	2015	82	3611	50.9	13.9 (0.9)
	Guyana	2010	76	1973	48.6	14.1 (0.8)
	Honduras	2012	79	1486	46.1	13.6 (1.0)
	India	2007	83	7330	57.4	13.9 (0.9)
	Indonesia	2015	94	8806	49.2	13.5 (1.0)
	Jordan	2007	99.8	1648	47.3	14.3 (0.7)
	Kiribati	2011	85	1340	45.5	14.0 (0.9)
	Laos	2015	70	1644	47.8	14.5 (0.8)
	Maldives	2009	80	1981	47.9	14.4 (0.7)
	Mauritania	2010	70	1285	53.2	14.2 (0.9)
	Mongolia	2013	88	3707	49.4	13.7 (1.0)
	Morocco	2016	91	3975	50.9	13.6 (1.1)
	Myanmar	2016	86	2237	46.3	13.6 (0.9)
	Northern Macedonia	2007	93	1550	51.6	13.9 (0.9)
	Pakistan	2009	76	4998	60.8	14.1 (0.8)
	Philippines	2015	79	6162	48.1	13.9 (0.9)
	Samoa	2011	79	2200	47.4	14.0 (0.8)
	Solomon Islands	2011	85	925	52.1	14.1 (0.9)
	Sri Lanka	2016	89	2254	49.3	13.9 (0.9)
	Sudan	2012	77	1401	51.9	14.2 (0.8)
	Syria	2010	97	2929	51.2	13.6 (1.0)
	Tunisia	2008	83	2549	49.7	13.6 (1.0)
	Vanuatu	2016	57	1288	47.8	14.1 (0.9)
	Vietnam	2013	96	1743	46.6	14.5 (0.6)
	Yemen	2014	75	1553	56.3	13.8 (1.0)
Upper middle	Algeria	2011	98	3484	45.8	13.6 (1.1)
	Antigua & Barbuda	2009	67	1235	51.4	13.9 (0.9)
	Argentina	2012	71	21,528	47.7	13.9 (0.9)
	Botswana	2005	95	1397	46.2	14.3 (0.8)
	Costa Rica	2009	72	2265	49.6	14.0 (0.9)
	Dominican Republic	2016	63	954	48.6	14.3 (1.0)
	Grenada	2008	78	1299	42.7	13.7 (1.1)
	Iraq	2012	88	1533	54.7	13.9 (1.0)
	Lebanon	2017	82	3347	47.4	13.6 (1.0)
	Libya	2007	98	1891	49.2	13.6 (1.0)
	Malaysia	2012	89	16,273	49.5	14.0 (0.9)
	Mauritius	2017	84	1955	45.8	13.9 (0.8)
	Namibia	2013	89	1936	42.9	14.1 (0.9)
	Paraguay	2017	87	1972	47.5	13.9 (1.0)
	Peru	2010	85	2359	49.9	14.1 (0.8)
	St. Lucia	2007	82	1072	44.5	13.7 (1.1)
	St. Vincent & the Grenadines	2007	84	1188	46.2	13.5 (1.0)
	Suriname	2016	83	1453	46.1	13.8 (1.0)
	Thailand	2015	89	4132	49.6	13.7 (1.0)
	Tonga	2017	90	2067	51.4	13.6 (1.1)
	Tuvalu	2013	90	679	48.9	13.3 (1.1)
High	Bahamas	2013	78	1308	47.3	13.4 (1.0)
	Barbados	2011	73	1504	51.1	14.1 (0.8)
	Brunei Darussalam	2014	65	1824	48.2	14.0 (0.9)
	Cayman Islands	2007	79	1147	52.0	13.5 (1.0)
	Curaçao	2015	83	1498	49.8	13.9 (1.1)
	French Polynesia	2015	70	1902	49.7	13.7 (1.0)
	Kuwait	2015	78	2034	49.4	14.1 (0.9)
	Oman	2015	92	1669	47.1	14.2 (0.8)
	Qatar	2011	87	1781	47.2	13.4 (1.0)
	St. Kitts & Nevis	2011	70	1471	50.2	14.1 (0.8)
	Trinidad & Tobago	2017	89	2763	48.3	13.6 (1.1)
	United Arab Emirates	2016	80	3471	48.1	13.9 (1.0)
	Uruguay	2012	77	2869	46.3	14.1 (0.8)

Abbreviation: SD Standard deviation; ^a^ Based on sample aged 12–15 years.

**Table 2:** Prevalence of never/rarely washing hands before eating, after using toilet, and with soap by country.

Country-Income	Country	%	95%CI	%	95%CI	%	95%CI
Low	Afghanistan	6.0	[4.4,8.2]	5.6	[3.6,8.6]	11.6	[8.0,16.6]
	Benin	6.4	[4.3,9.2]	10.6	[5.6,19.2]	18.5	[13.2,25.3]
	Cambodia	2.1	[1.5,3.0]	3.0	[2.2,4.2]	2.9	[2.2,3.7]
	Kenya	8.6	[7.0,10.5]	13.9	[11.9,16.1]	16.6	[14.1,19.5]
	Liberia	12.3	[9.2,16.4]	6.3	[4.1,9.6]	9.6	[6.9,13.3]
	Malawi	4.1	[2.2,7.6]	5.3	[3.3,8.4]	16.7	[13.5,20.4]
	Mozambique	7.4	[4.9,11.1]	7.8	[4.9,12.1]	11.7	[7.1,18.7]
	Nepal	4.0	[2.8,5.7]	4.4	[3.3,5.8]	4.8	[3.8,6.2]
	Senegal	10.2	[4.9,20.0]	10.0	[4.8,19.7]	14.7	[9.4,22.4]
	Tanzania	7.3	[6.1,8.7]	17.3	[14.3,20.8]	20.7	[18.2,23.4]
	Uganda	6.3	[5.0,8.0]	8.0	[6.1,10.5]	14.7	[12.2,17.7]
	Zambia	12.5	[10.3,15.1]	15.4	[12.9,18.3]	20.8	[18.1,23.8]
Lower middle	Bangladesh	3.1	[1.8,5.2]	1.9	[0.8,4.4]	5.0	[2.8,8.6]
	Belize	3.7	[3.1,4.4]	1.6	[1.2,2.2]	4.6	[3.6,5.8]
	Bolivia	10.8	[9.4,12.4]	7.3	[6.1,8.7]	16.0	[13.8,18.5]
	Djibouti	6.0	[4.6,7.7]	12.9	[11.4,14.6]	11.7	[9.4,14.5]
	East Timor	20.9	[18.8,23.2]	27.5	[24.5,30.8]	18.9	[16.4,21.6]
	Egypt	12.9	[10.5,15.7]	9.3	[6.9,12.5]	7.8	[5.7,10.6]
	El Salvador	5.3	[4.1,6.8]	4.0	[2.7,6.1]	5.7	[4.5,7.3]
	Eswatini	3.6	[2.3,5.5]	2.8	[2.3,3.5]	11.6	[10.0,13.4]
	Ghana	7.5	[4.6,12.2]	7.8	[5.0,11.8]	10.6	[8.6,13.1]
	Guatemala	4.5	[2.8,7.1]	2.7	[1.5,4.7]	5.7	[4.2,7.6]
	Guyana	9.2	[7.4,11.3]	6.4	[4.8,8.4]	10.7	[8.5,13.3]
	Honduras	7.5	[6.4,8.7]	5.2	[4.0,6.7]	58.6	[54.9,62.3]
	India	5.9	[5.0,7.1]	3.3	[2.7,4.1]	13.0	[11.4,14.7]
	Indonesia	2.5	[2.0,3.0]	2.3	[1.8,2.9]	3.9	[3.3,4.7]
	Jordan	6.8	[5.5,8.3]	7.0	[5.0,9.7]	9.2	[7.4,11.5]
	Kiribati	20.6	[16.3,25.7]	16.1	[13.1,19.5]	15.4	[13.2,17.9]
	Laos	1.9	[1.1,3.2]	3.8	[2.5,5.9]	8.5	[6.7,10.7]
	Maldives	8.5	[7.1,10.1]	4.7	[3.7,5.8]	7.2	[5.9,8.8]
	Mauritania	7.6	[6.2,9.2]	24.4	[17.8,32.4]	12.1	[9.4,15.6]
	Mongolia	6.9	[5.9,8.0]	10.5	[8.9,12.3]	3.0	[2.4,3.8]
	Morocco	4.8	[4.1,5.5]	6.2	[5.1,7.6]	10.4	[8.6,12.5]
	Myanmar	6.7	[5.5,8.2]	8.4	[6.8,10.4]	5.8	[4.7,7.1]
	Northern Macedonia	2.1	[1.5,3.0]	2.1	[1.4,3.2]	3.8	[2.7,5.3]
	Pakistan	3.4	[2.7,4.4]	3.4	[2.5,4.6]	8.0	[6.3,10.2]
	Philippines	7.7	[4.9,11.8]	6.3	[3.9,10.0]	7.9	[5.4,11.5]
	Samoa	14.6	[12.9,16.4]	17.1	[14.9,19.5]	19.3	[17.1,21.7]
	Solomon Islands	8.6	[7.2,10.2]	8.9	[6.9,11.5]	10.3	[7.4,14.0]
	Sri Lanka	2.7	[1.9,3.9]	3.0	[2.1,4.1]	7.1	[5.6,9.0]
	Sudan	7.5	[6.0,9.4]	11.9	[9.9,14.1]	20.9	[16.7,25.9]
	Syria	9.2	[7.5,11.3]	3.8	[2.5,5.8]	6.7	[5.1,8.8]
	Tunisia	6.0	[5.2,6.9]	4.0	[3.2,4.9]	4.7	[3.7,5.9]
	Vanuatu	4.2	[3.3,5.5]	5.1	[3.9,6.6]	6.4	[5.1,8.1]
	Vietnam	7.0	[5.4,9.0]	2.2	[1.5,3.3]	8.5	[6.5,10.9]
	Yemen	7.2	[5.1,10.1]	11.2	[9.6,13.0]	15.3	[12.7,18.4]
Upper middle	Algeria	5.6	[4.9,6.4]	3.1	[2.5,3.8]	5.1	[4.4,5.8]
	Antigua & Barbuda	12.2	[10.5,14.2]	5.1	[3.9,6.7]	7.1	[5.7,8.9]
	Argentina	11.2	[10.1,12.4]	5.9	[5.0,7.0]	6.2	[5.5,7.1]
	Botswana	4.5	[3.3,6.1]	5.1	[4.3,6.2]	17.0	[13.9,20.7]
	Costa Rica	9.6	[8.3,11.1]	2.2	[1.7,2.8]	5.4	[4.5,6.5]
	Dominica	14.1	[10.1,19.2]	5.3	[3.7,7.7]	8.6	[5.8,12.6]
	Grenada	11.4	[9.5,13.6]	3.5	[2.8,4.4]	10.1	[8.4,12.1]
	Iraq	7.3	[5.6,9.6]	8.1	[6.3,10.3]	4.0	[3.0,5.4]
	Lebanon	4.1	[3.3,5.0]	1.6	[1.1,2.4]	2.2	[1.9,2.7]
	Libya	8.1	[6.3,10.4]	6.8	[5.5,8.5]	7.4	[5.9,9.1]
	Malaysia	5.0	[4.5,5.6]	6.0	[5.3,6.7]	13.6	[12.7,14.6]
	Mauritius	13.9	[11.1,17.4]	4.0	[2.7,6.0]	8.2	[6.1,10.8]
	Namibia	5.5	[4.4,6.8]	6.4	[5.1,8.0]	10.8	[8.8,13.1]
	Paraguay	8.0	[6.6,9.7]	2.6	[1.9,3.5]	6.1	[5.0,7.4]
	Peru	4.9	[3.8,6.2]	6.6	[5.5,7.9]	7.5	[6.0,9.4]
	St. Lucia	16.5	[14.0,19.3]	4.4	[3.4,5.6]	11.1	[9.1,13.4]
	St. Vincent & the Grenadines	8.7	[6.7,11.3]	4.1	[3.0,5.5]	8.3	[6.6,10.3]
	Suriname	11.2	[8.8,14.2]	4.3	[3.0,6.1]	8.1	[6.7,9.9]
	Thailand	14.9	[12.5,17.7]	6.5	[5.4,7.9]	14.4	[12.3,16.9]
	Tonga	14.9	[12.9,17.0]	7.8	[6.5,9.4]	20.2	[18.2,22.4]
	Tuvalu	38.5	[34.9,42.4]	17.7	[15.0,20.8]	18.0	[15.2,21.0]
High	Bahamas	15.6	[13.5,18.0]	4.5	[3.4,5.8]	8.7	[7.2,10.4]
	Barbados	14.3	[11.9,17.1]	1.7	[1.2,2.5]	7.9	[6.4,9.7]
	Brunei Darussalam	3.5	[2.7,4.5]	2.9	[1.9,4.4]	9.7	[8.1,11.5]
	Cayman Islands	10.8	[9.1,12.8]	4.4	[3.4,5.8]	5.9	[4.7,7.5]
	Curaçao	12.2	[10.2,14.4]	3.6	[2.6,4.8]	6.5	[5.1,8.3]
	French Polynesia	8.4	[6.5,10.7]	5.7	[4.2,7.8]	11.5	[9.8,13.6]
	Kuwait	10.7	[9.0,12.7]	6.2	[4.8,8.0]	7.4	[5.6,9.8]
	Oman	7.9	[5.9,10.4]	8.8	[6.8,11.2]	7.4	[5.6,9.8]
	Qatar	17.0	[13.8,20.8]	15.1	[12.3,18.4]	16.4	[13.0,20.4]
	St. Kitts & Nevis	11.6	[10.1,13.4]	2.9	[2.1,3.9]	6.7	[5.5,8.1]
	Trinidad & Tobago	14.7	[12.9,16.7]	3.5	[2.2,5.5]	11.7	[9.4,14.5]
	United Arab Emirates	8.1	[6.9,9.6]	3.2	[2.5,4.3]	4.3	[3.5,5.3]
	Uruguay	12.5	[10.8,14.5]	6.9	[5.5,8.5]	3.6	[2.7,4.8]

Abbreviation: CI Confidence interval.

**Table 3:** Association between severe food insecurity and never/rarely washing hands before eating, after using toilet, and with soap estimated by meta-analysis with random effects based on country-wise estimates.

Outcome	Country-Income	OR (95%CI)	*I*^2^ (%)
Before eating	Low	1.36 [1.12,1.66]	14.8
	Lower middle ^a^	1.29 [1.17,1.43]	50.8
	Upper middle	1.39 [1.22,1.57]	59.7
	High	1.38 [1.17,1.62]	45.7
	Overall	1.34 [1.25,1.43]	48.2
After using toilet	Low	1.42 [1.19,1.70]	54.8
	Lower middle	1.49 [1.34,1.66]	35.8
	Upper middle	1.82 [1.58,2.09]	51.3
	High	2.00 [1.62,2.49]	0.0
	Overall	1.61 [1.50,1.73]	41.3
Soap	Low	1.34 [1.17,1.52]	62.8
	Lower middle ^b^	1.26 [1.13,1.40]	50.4
	Upper middle	1.63 [1.45,1.84]	67.7
	High	1.82 [1.52,2.18]	0.0
	Overall	1.44 [1.35,1.53]	57.4

Abbreviation: OR Odds ratio; CI Confidence interval; Country-wise estimates were adjusted for age and sex; ^a^ Laos was not included because estimates could not be obtained due to small numbers; ^b^ Laos and Northern Macedonia were not included because estimates could not be obtained due to small numbers.

## Data Availability

The data that support the findings of this study are available from the corresponding author, upon reasonable request.
